# Paget’s Disease of Bone and Chronic Kidney Disease: A Review

**DOI:** 10.3390/jcm15082843

**Published:** 2026-04-09

**Authors:** Lorena Traversari

**Affiliations:** Nephrology and Dialysis Unit, S. Andrea Hospital, Risorgimento Avenue 43, 58024 Massa Marittima, Italy; lorena.traversari@uslsudest.toscana.it

**Keywords:** Paget’s disease of bone, chronic kidney disease, survival, mortality, kidney transplantation

## Abstract

**Introduction:** Paget’s disease of bone (PDB), the second most common bone disease after osteoporosis, is still of unknown etiology and thus deserves attention. PDB and chronic kidney disease (CKD) are chronic diseases characterized by alterations in bone turnover, mineralization, volume, and strength. Both conditions carry an increased cardiovascular risk, as well as increased morbidity and mortality. Both are common in the Western world and primarily affect men over 50 years of age. Despite these similarities, little data exists on their coexistence. **Purpose and Methodology:** By evaluating the available literature, we found extensive documentation on individual diseases, which has led to consolidated guidelines. The coexistence of the two diseases has provided sporadic studies describing individual cases or small case series. More limited information is available on patients who have received or are eligible for kidney transplants and also have PDB. This narrative (non-systematic) review aims to examine the topic with a particular focus on the relationship between PDB and CKD, especially concerning issues around kidney transplantation. The overlapping factors of the two diseases, and their impact on PDB diagnosis and treatment are discussed. Additionally, examining CKD patients may offer valuable insights for the design of prospective longitudinal or cross-sectional studies aimed at expanding our understanding of PDB. **Limitations:** The different points of discussion that emerged from the examination of this topic may be useful in the management of PDB-CKD patients but, at the moment, there are not enough data available to draw definitive conclusions to support clinical practice. **Conclusions and Future Directions:** The coexistence of PDB and CKD is not a rare phenomenon; studying patients with both diseases could provide insights into new research avenues. Above all, and more immediately, attention to the coexistence of the two diseases could improve patient management with personalized choices based on their renal function.

## 1. Introduction

Paget’s disease of bone (PDB), described in 1877, remains a clinical entity of unknown etiology, despite being the second most common bone disease after osteoporosis [1]. This review summarizes the current knowledge on the etiology, prevalence, diagnosis, and treatment of PDB, with emphasis on the limited data regarding the coexistence of PDB and chronic kidney disease (CKD). Beyond the simple epidemiological coincidence, identifying the coexistence of both diseases would allow for personalized clinical therapeutic treatment aimed at avoiding complications linked to the use of nephrotoxic drugs and/or drugs excreted by the kidneys. The complex therapeutic decisions that should be made when both diseases are present, along with their complications, are also explored. Furthermore, studies on this topic could provide useful data to understand whether, to what extent, and how PDB can influence the outcomes of patients with CKD and vice versa.

## 2. Purpose and Methodology

By evaluating the studies available in the literature, we aim to highlight aspects of the coexistence of PDB and CKD that may stimulate interest in conducting prospective studies and/or to strengthen multicenter registries with data that pertains to both pathologies and that can be used to provide new information, evidence, and recommendations. The keywords listed above were used to identify the cited bibliographic sources using PubMed, Google Scholar, and Google. The methodological approach to addressing the current knowledge on PDB and CKD is based on the latest published guidelines for the management of the two diseases. National and international studies were selected to obtain information on the prevalence and distribution of PDB worldwide. These studies, supplemented by reviews of PDB published at different times and obtained from the same search engines, were used to compile the information reported in the Section 4 and Section 5. For greater completeness, additional information was obtained from clinical case studies and by carefully examining the references of the selected works. For greater clarity, the exposition of the studies reported in the text is accompanied by round brackets indicating the number of patients observed and/or other useful information. The search did not provide data on the coexistence of the two diseases; therefore, the keywords PDB and CKD were used simultaneously, identifying studies reporting clinical cases or case series that provided interesting points for discussion. The search engines used highlighted a gap in the available literature regarding the coexistence of the two conditions and patient management. Furthermore, no studies or clinical cases involving renal transplant patients with PDB were found. The lack of data led to the conclusion that a review of the topic would be useful, as it could foster interest and encourage new studies. The first part of this paper summarizes the information regarding PDB, then it introduces the relationship between PDB and CKD, and, finally, it explores the issues raised by the management of patients with the two diseases and the implications of PDB for renal transplantation. At the end of each section, which encourages questions or doubts, research questions and related considerations are presented, highlighting the lack of sufficient data to develop evidence-based strategies or conclusions.

## 3. Etiology and Epidemiology

The cause of PDB remains unknown despite extensive research efforts aimed at uncovering its origins. In a 2022 review, Gennari L. et al. summarized the recent findings, exploring genetic, environmental, and viral factors and highlighting the potential role of epigenetics, which may explain the incomplete penetrance in familial cases of PDB [2]. The pathogenesis appears multifactorial, with germline or somatic mutations and epigenetic mechanisms potentially interacting with viral (e.g., paramyxoviruses) and environmental (e.g., pesticides, heavy metals, and tobacco exposure) factors [2]. A study linked to the General Practice Research Database (GPRD) in England and Wales and a meta-analysis showed that PDB is more frequent in white males over 50 years of age and certain ethnic groups [3,4]. The highest prevalence is reported in the United Kingdom, Australia, and New Zealand, followed by the United States and Canada, while it is uncommon in Asia. This geographical distribution suggests that PDB originated in Europe and may have spread worldwide through migration (109 cases) [5]. Epidemiological research in Italy began in the 1960s (quoted by L. Gennari 2002) [6], and, in the 1980s, cities such as Milan and Palermo contributed to an international study alongside other European cities [7]. In the 2000s, Italy established a National Registry for PDB and conducted studies in Turin and Siena [8]. The findings estimate that the national prevalence is at least 1%, with more males affected than females. Unlike high-prevalence countries (e.g., the UK, Australia, and New Zealand), Italy has seen no reduction in PDB prevalence or severity in recent decades [8]. In Italy, PDB distribution is not uniform; the highest prevalence and severity are reported in Campania, a southern region, where earlier onset, increased severity, and higher neoplastic degeneration in familial forms are also observed (236 cases) [9].

### Research Questions and Considerations Related to the Frequency of CKD in Patients with PDB

1. What is the prevalence of chronic kidney disease (CKD) in patients with PDB?
2. Do national and international studies conducted to determine the prevalence of the disease in various countries provide information on the frequency of CKD in the patients examined? Would this information be accessible for conducting retrospective studies?

## 4. Features, Clinical Presentation, and Complications

PDB is a chronic metabolic disorder characterized by abnormal bone remodeling, with excessive resorption followed by increased bone formation. Although the newly formed bone is abundant, it has structural abnormalities that make it weak and prone to fractures and deformities. Affected areas are clearly demarcated from healthy bone, and, although any bone may be impacted, the pelvis, spine, skull, femur, and tibia are the most commonly involved (as reported in 889 patients) [10]. The disease may affect one site (monostotic form) or multiple sites (polyostotic form), with an asymmetrical distribution. Pagetic bone typically appears enlarged and deformed, probably due to dysfunction of the osteoclasts, which are both larger and more numerous, with each containing multiple nuclei [11,12]. The presence of inclusion bodies has been described, which can be explained by abnormalities in the cellular machinery due to misfolded proteins or abnormalities in proteasomal functions or autophagy pathways [13]. Abnormalities in cellular autophagy mechanisms promote metabolic hyperactivity in these areas, which also present increased vascularity. Bone weakness and deformation in PDB can lead to complications, including hearing loss, obstructive hydrocephalus, and spinal canal stenosis. Spinal stenosis may progress to paraplegia, which also results from a vascular “steal phenomenon” caused by high blood demand from hypermetabolic bone areas. This hypothesis was suggested following the observation of 8 + 19 cases of paraparesis treated with prolonged medical therapy (calcitonin or bisphosphonates) rather than surgery, where a favorable clinical response was reported in approximately 90% of patients [14]. Symptoms and neurological complications vary depending on the bones involved and the compression of the peripheral or cranial nerves; for example, headache has also been described [15,16]. Additionally, bone fragility and deformity elevate the risk of fractures, particularly in the femur and vertebrae [17]. Lesions near joints contribute to osteoarthritis, with increased surgical needs for hip and knee replacements (as reported in a study linked to the GPRD) [18]. Neoplastic transformation is rare (under 1%, based on data obtained from the observation of 3964 patients), with reported cases of osteosarcoma, fibrosarcoma, chondrosarcoma, and giant cell tumors (in a study involving the Italian PDB Registry and a PRISMA study conducted in the UK) [19,20]. Although metastases from cancers such as lymphoma, prostate cancer, and breast cancer occur in patients with PDB, they rarely form in pagetic bone, suggesting that PDB-specific bone alterations may not support metastatic cell growth (3 + 26 cases) [21].

## 5. Extraosseous Complications

Additional complications of PDB include kidney stones and cardiovascular manifestations, such as cardiac valve calcifications, vascular calcifications, and high-output heart failure (reported in an Italian study on 147 patients with PDB versus 323 non-PDB controls) [22]. The GPRD study in England and Wales found that patients with PDB had a higher 5-year mortality rate (32.7%) than controls (28%) [3]. An observational cross-sectional study in 54 PDB patients and 54 non-PDB controls revealed increased fasting glucose levels and elevated systolic blood pressure in PDB patients compared to controls. The authors observed a positive correlation between glucose levels and ionized calcium/bone-specific alkaline phosphatase (BALP), as well as systolic hypertension and elevated alkaline phosphatase (ALP)/BALP; they hypothesized that these findings could indicate a link to cardiometabolic changes [23]. Studies continue to confirm a higher prevalence of impaired glucose metabolism and hypertension in PDB patients, and, while current data comes from studies with small cohorts (to exemplify, we cite a cross-sectional, case–control study with 23 PDB patients and 30 healthy controls), broadening clinical evaluations for cardiovascular risk in PDB patients is advisable [24].

Hypercalcemia, hypercalciuria, hyperoxaluria, and hyperuricemia are possible metabolic complications that may lead to kidney stone formation, the most frequent renal alteration associated with PDB. PDB is a risk factor for nephrolithiasis (NL), regardless of disease activity, with NL being more prevalent in polyostotic cases (as reported in a cross-sectional multicentric study involving 97 PDB patients with NL, 219 PDB patients without NL, 364 NL patients without PDB, and 219 controls) [25]. As such, it may be prudent to screen for PDB in NL assessments in middle-aged and older adults (864 PDB patients versus 500 controls) [26]. Hormonal alterations in PDB were identified as early as the 1980s, when secondary hyperparathyroidism (sHPT) was observed in 12–18% of untreated PDB patients. This finding may be an expression of the disease (independent of treatment) and appears unrelated to disease spread or activity. A rise in PTH may result from the increased calcium demand associated with pagetic bone formation (reported in one study on 151 PDB patients and one study on 39 PDB patients) [27,28]. Increased PTH could stimulate remodeling in healthy bone areas (as observed in transiliac bone biopsies from 136 untreated PDB patients, with 72 biopsy samples from pagetic areas and 64 biopsy samples from non-pagetic areas) [29]. It remains unclear whether healthy bone remodeling, with its associated calcium release, may encourage further pagetic bone formation and whether elevated PTH may amplify osteoclast activation in pagetic regions [30]. sHPT can arise from hypocalcemia due to calcium and/or vitamin D deficiencies, which may result from antiresorptive treatments, such as bisphosphonates (as reported in studies examining small numbers of patients: 40 + 63 + 18 + 1 cases), which are now the primary therapeutic option [31,32,33,34,35]. During antiresorptive therapy, maintaining normal calcium levels with calcium and vitamin D supplements is essential [32]. Although the coexistence of primary hyperparathyroidism (pHPT) and PDB has also been reported, there is no etiological connection between the two diseases; thus, this association may simply be coincidental, only reflecting the co-occurrence of two common age-related conditions [30]. In patients treated with parathyroidectomy, PDB should be excluded if PTH remains elevated post-surgery [30].

## 6. Diagnosis

Simple and affordable tests allow for PDB diagnosis, but many cases remain undiagnosed due to prolonged asymptomatic periods; less than 10% of patients with radiological signs of PDB seek medical attention, meaning that the frequency of extraosseous complications is largely unknown [3]. The most common symptom of PDB is bone pain, which does not correlate with disease activity, as over 40% of patients with elevated ALP levels report no pain (data obtained from two clinical studies: Reid evaluated 55 PDB patients, and Langston evaluated a cohort of 1324 PDB patients) [36,37]. Bone pain often prompts patients to seek medical attention, leading to diagnosis, though the disease is frequently discovered incidentally during routine tests. Characteristic bone alterations that aid diagnosis include osteolytic areas, cortical and trabecular thickening, indistinct cortex–medulla boundaries, osteosclerosis, bone expansion, and bone deformity [35]. While each of these findings may be non-specific alone, they are diagnostic in combination [35]. Although any bone segment may be involved, radiological screening is effective in detecting PDB in 93% of cases if it includes plain X-rays of the abdomen, tibias, skull, and facial bones (208 patients studied) [38]. Radionuclide total bone scintigraphy (Tc99m) is recommended to assess the extent and distribution of active disease [39]. To evaluate bone complications, such as osteosarcoma or medullary canal stenosis, magnetic resonance imaging (MRI) and/or computed tomography (CT) are invaluable (13 cases) [40]. ALP is among the most studied markers of bone turnover, and its presence in blood and urine makes it widely accessible and cost-effective. During diagnosis, it is important to recognize that ALP is not specific to Paget’s disease, as only 42% of patients with radiographically confirmed lesions show elevated levels (as reported in the Rotterdam Study—a prospective cohort study based on a population over 54 years of age) [41]. Elevated ALP levels with normal liver function should prompt testing for BALP to confirm bone involvement. BALP is particularly useful when ALP levels are normal, as it may still be elevated in more than half of individuals with normal ALP levels and confirmed PDB (59 cases) [42]. Other bone turnover markers, such as collagen peptides, have been studied, though no significant differences were found between ALP, BALP, and collagen peptide levels when assessing the presence and extent of pagetic bone changes (meta-analysis) [43].

## 7. Medical Therapy Strategies

There have been no updates to therapeutic management guidelines since the 2019 UK Guidelines, which recommend bisphosphonates as the first-line treatment for PDB, based on findings from randomized controlled trials [35,44]. Zoledronic acid shows superior efficacy in managing bone pain compared to pamidronate, alendronate, and risedronate [35]. Currently, no evidence supports the idea that bisphosphonates improve quality of life, prevent fractures, slow hearing loss progression, or prevent/treat deformities associated with PDB [35,44]. Bisphosphonates in general and nitrogen-containing bisphosphonates (NCBs) in particular effectively reduce bone turnover, as evidenced by ALP normalization. Among NCBs, zoledronic acid is most effective, showing longer-lasting control over ALP levels when administered as a single 5 mg intravenous dose, maintaining ALP normalization for up to 5 years [35]. Bisphosphonate treatment aims to control bone pain and normalize ALP. The results of treating asymptomatic patients to prevent complications remain inconclusive. For patients unable to use bisphosphonates, calcitonin provides an alternative for short-term pain relief and ALP reduction, though it is more costly and linked to an increased risk of cancer with long-term use; thus, it is recommended only for short-term use [35]. Denosumab has no current support as an effective treatment for PDB, so its use is not advised [35]. Anabolic agents, such as teriparatide, are contraindicated for PDB, even after the FDA’s removal of the boxed warning of osteosarcoma risk [45,46]. For pain management in symptomatic patients, analgesics, non-steroidal anti-inflammatory drugs (NSAIDs), and anti-neuropathic medications are commonly prescribed, though none have been specifically studied for PDB pain management [35].

## 8. Follow-Up

Monitoring is particularly important for PDB patients who have received antipagetic therapy, with an initial assessment of response after three and six months via ALP measurements. If results are satisfactory, semi-annual or annual monitoring may suffice (practice guidelines) [47,48]. Additionally, annual radiographic evaluations are recommended for osteolytic lesions to assess for improvement or worsening following treatment. In asymptomatic, untreated patients, regular ALP monitoring helps evaluate disease progression, even in monostotic cases or cases with normal ALP levels. BALP testing can also assist in such evaluations [48].

## 9. Prognosis

**Figure 1:**
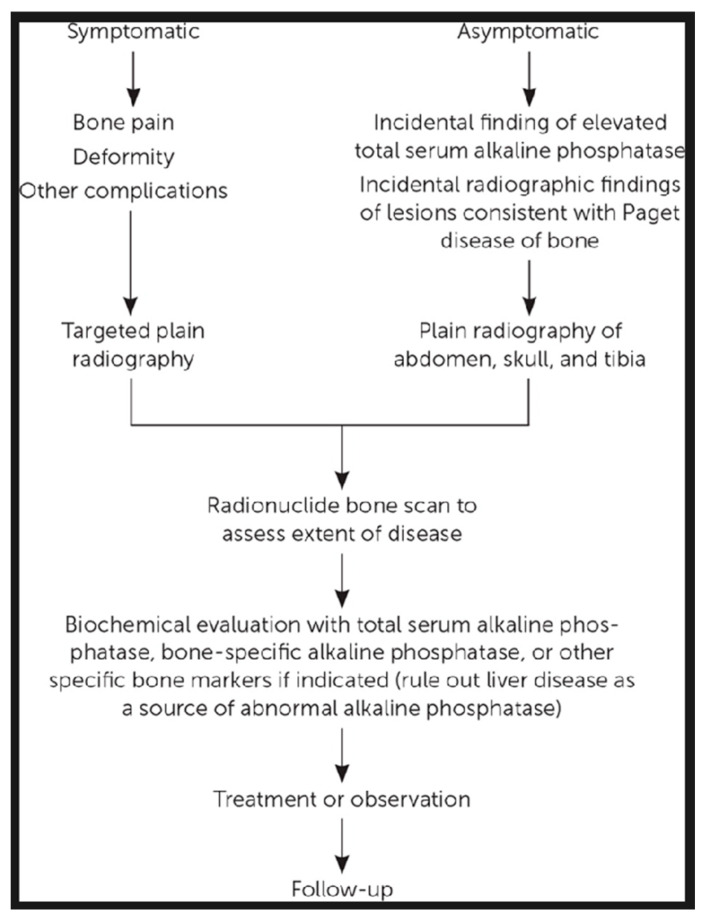
Diagnosis and management of Paget disease of bone, as recommended by J.R. Nahid and K. Jude [49].

**Figure 2:**
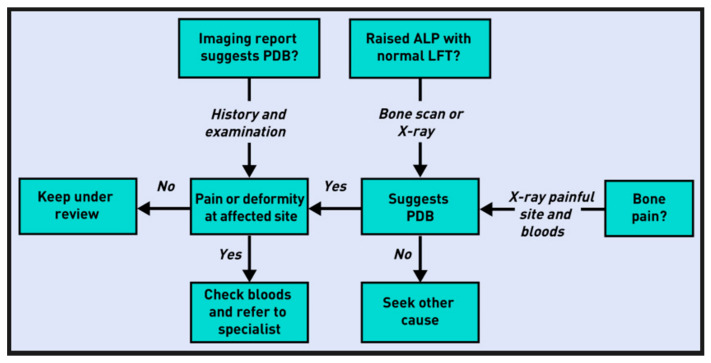
Diagnosis and referral of patients suspected of having PDB, as recommended by C. Nairn and S.H. Ralston [50].

Although the most recent guidelines (2019 UK Guidelines) indicate that there is no clear evidence that specific treatments slow disease progression or prevent complications, early diagnosis and access to treatments before severe complications develop may improve prognosis [35]. Early diagnosis as a basis for effective disease management has encouraged the dissemination of practical information for primary care published in 2020 in the UK and the US [49,50]. J.R. Naihid and K. Jude provide a diagnostic flowchart for suspected PDB cases, as shown in Figure 1 [49]; C. Nairn and S.H. Ralston offer guidance on referring patients to secondary care, as shown in Figure 2 [50]; and the American Academy of Family Physicians has released patient information titled “Paget Disease of Bone: What You Should Know,” available online at https://www.aafp.org. Although PDB incidence and severity appear to be decreasing in high-prevalence populations, interest in the disease persists. These shifts in epidemiology may warrant continued research to better understand its etiopathogenesis [51].

## 10. Discussion on the Relationship Between PDB and CKD

While the literature on PDB is extensive, information regarding PDB and CKD remains scarce, even in Western countries with high PDB prevalence.

The CARHES study (Cardiovascular Risk in Renal Patients of the Health Examination Survey) reported a CKD prevalence of 6.3% in Italy. According to data from the Italian Institute of Statistics (ISTAT) [52], in January 2025, the population aged over 50 years in the province of Grosseto (Tuscany—central Italy) numbered 111,252 people. The number of patients with PDB was determined to be approximately 1162, with 7323 cases of CKD, which suggests that, even in areas with a low population density, cases with the coexistence of both diseases can still be identified. Table 1 shows the simple calculation used to estimate the combined prevalence of the two diseases (considering independent variables). The result obtained is a prevalence of 0.063%. It can be estimated that there are at least 80 PDB-CKD patients in the Grosseto area. This calculation does not consider CKD cases potentially resulting from the complications of PDB. Because of the lack of data, a correct estimate cannot be made, though such data could be obtained from cross-sectional or cohort studies.

To date, only 16 cases involving both PDB and CKD have been documented (Table 2) [53,54,55,56,57,58,59,60,61,62,63,64,65,66]. A prospective study was conducted in 21 patients over seventy years of age undergoing dialysis, hemodialysis, or chronic ambulatory peritoneal dialysis (CAPD) to assess the presence of skeletal alterations. The study reported pagetic lesions in 5% of cases, along with other more frequent lesions [67].

The coexistence of these two conditions may simply reflect their prevalence in the population; however, in some cases, the complications of PDB can cause CKD. Long-lasting hypercalcemia, nephrolithiasis, and obstructive tubulopathy from crystal precipitation are potential causes of CKD in PDB patients. Hypercalcemia may result from immobility in severe cases or from vitamin D and calcium therapy. PDB itself is an independent risk factor for nephrolithiasis (NL), as metabolic remission (normal ALP) in PDB patients still doubles the incidence of stone formation compared to in controls [25]. Cardiovascular risk factors linked to PDB, such as arterial hypertension and valvular calcifications, increase the likelihood of heart failure, further complicating renal health [22].

sHPT is a common condition in CKD patients, even in the early stages of disease, and it may mask asymptomatic forms of PDB with ALP levels in the normal range since it induces the nephrologist not to proceed with further investigations, limiting the action to the control of sHPT by means of Vit D/Vit D analogs, calcimimetics and if necessary calcium supplements after adequate control of phosphatemia. Hypocalcemia and hyperphosphatemia, typical alterations of sHPT in the advanced stages of CKD, lead to cardiac and vascular calcifications [68]. These calcifications, which may also occur as a result of the therapy used to treat metabolic bone disease in CKD (CKD-MBD), contribute to reducing the survival of CKD patients [69,70]. Both PDB and CKD patients have increased mortality and vascular and cardiac calcifications as a result of abnormal bone remodeling.

### Research Questions and Considerations in Relation to the Presence of References in the Literature on the Coexistence of CKD in Patients with PDB

1. Why is this association seldom discussed?
2. Would a questionnaire regarding the coexistence of the two diseases addressed to general practitioners and nephrologists be of any use?
3. How many PDB patients develop CKD as a complication?
4. In the studies conducted, did the evaluation of sHPT data take into account the glomerular filtration rate (GFR) (by age, sex, and body weight) of patients with PDB? Is it possible that sHPT is partly due to the underestimated coexistence of early or moderate stages of CKD?

## 11. Pharmacological Management Challenges in CKD Patients with PDB

The presence of CKD complicates the treatment of PDB, as antipagetic drugs are primarily excreted through the kidneys via glomerular filtration and tubular secretion [71,72]. As CKD progresses, drug concentrations can increase in the blood and bone, resulting in prolonged and excessive skeletal effects. This may worsen CKD-MBD and depress bone turnover, leading to conditions such as osteomalacia, mixed uremic osteodystrophy, and adynamic bone disease (ABD) [73]. Bisphosphonate nephrotoxicity, especially from intravenous administration, is known to potentially induce acute renal failure (ARF) due to glomerular sclerosis and acute tubular necrosis. Recovery from ARF is variable, with some patients experiencing partial, full, or no recovery of renal function after stopping the drug (as reported in one review and three cases) [72,74]. Nephrotoxicity risk is dose-dependent and influenced by the infusion rate; hence, spacing out doses is recommended [75]. Given these risks, bisphosphonates are generally contraindicated when GFR falls below 35–30 mL/min/1.73 m^2^ [72,75]. Research indicates that osteoporosis treatments with bisphosphonates are managed similarly in CKD and non-CKD patients in primary care, despite contraindications for low GFR and the specifics of CKD-MBD [76].

For dialysis patients, a new avenue might become available, as clodronate, pamidronate, and ibandronate are dialyzable and may be feasible options, though dedicated studies on this are needed [77]. The latest KDIGO guidelines on CKD-MBD recommend considering both biochemical abnormalities and CKD progression in treatment decisions, with a possible role for bone biopsy in G3a-G5D CKD patients presenting with low bone mineral density (BMD) and/or fragility fractures; routine measurement of bone turnover markers derived from collagen synthesis is not suggested [78]. NSAIDs are also advised against in CKD patients, as they can accelerate GFR decline, worsen electrolyte imbalance and fluid overload, and exacerbate coexisting hypertension and heart failure [79]. Combining NSAIDs with diuretics or renin–angiotensin system inhibitors (RASIs) may further reduce GFR by affecting glomerular hemodynamics (as reported in a retrospective review of clinical/administrative records) [80]. Calcium and vitamin D supplements used for both conditions require careful monitoring to avoid hypercalcemia, as reported in the KDIGO guidelines [78].

Table 3 lists the pharmacological precautions for patients with CKD.

The complex pharmacological approach to PDB, which is due to the contraindications of bisphosphonates in CKD for GFR < 35–30 mL/min/1.73 m in isolated cases (listed in Table 2), has led to the use of denosumab, a drug not mentioned in the guidelines, with reported positive results; however, the data should be considered anecdotal.

## 12. Considerations for CKD-MBD and Bone Turnover in PDB and CKD Patients

Determining the presence or absence of CKD-MBD in patients with both osteoporosis and CKD is crucial, as it helps to differentiate renal osteodystrophy types with high or low bone turnover [81]. Likewise, identifying CKD-MBD in patients with both CKD and PDB may be necessary. Assessing non-pagetic bone for CKD-related changes may help distinguish among renal osteodystrophy types, with histomorphometric characteristics identifiable by bone biopsy using the TMV classification, which assesses turnover, mineralization, and volume (practice guidelines) [82]. Knowledge of CKD-MBD is changing: initial studies showed predominant alterations related to high bone turnover. In more recent studies, an increase in normal bone turnover was reported. However, major findings from bone biopsies performed in the USA and Europe (as well as in Turkey, Venezuela, and Brazil) showed lesions from low turnover. Furthermore, an increasing presence of osteoporosis has been documented, which complicates the already complex pathogenesis of CKD-MBD [83].

Table 4 summarizes the histological changes observed in the various types of renal osteodystrophy.

Kidney transplantation does not solve the problems faced by CKD patients, as the permanence, progression, or evolution of lesions into other types has been documented independently of renal function. Osteoporosis has also been described in post-transplant patients. The causes of these changes could be accredited, at least in part, to the therapies used for CKD-MBD and kidney transplantation [83].

In CKD G3-G5D patients, bone turnover can be evaluated based on PTH and BALP levels, which help distinguish high or low bone turnover patterns (practice guidelines) [84]. Because routine biopsies are impractical due to their limited availability and high costs, these biochemical markers can provide insights into bone turnover patterns in patients with CKD and PDB. Importantly, the KDIGO guidelines recommend measuring serum calcium, phosphate, PTH, and ALP levels in CKD patients with GFR < 45 mL/min/1.73 m^2^ at least once, making this group, in our opinion, also suitable for PDB diagnostic screening (Table 5).

**Figure 3:**
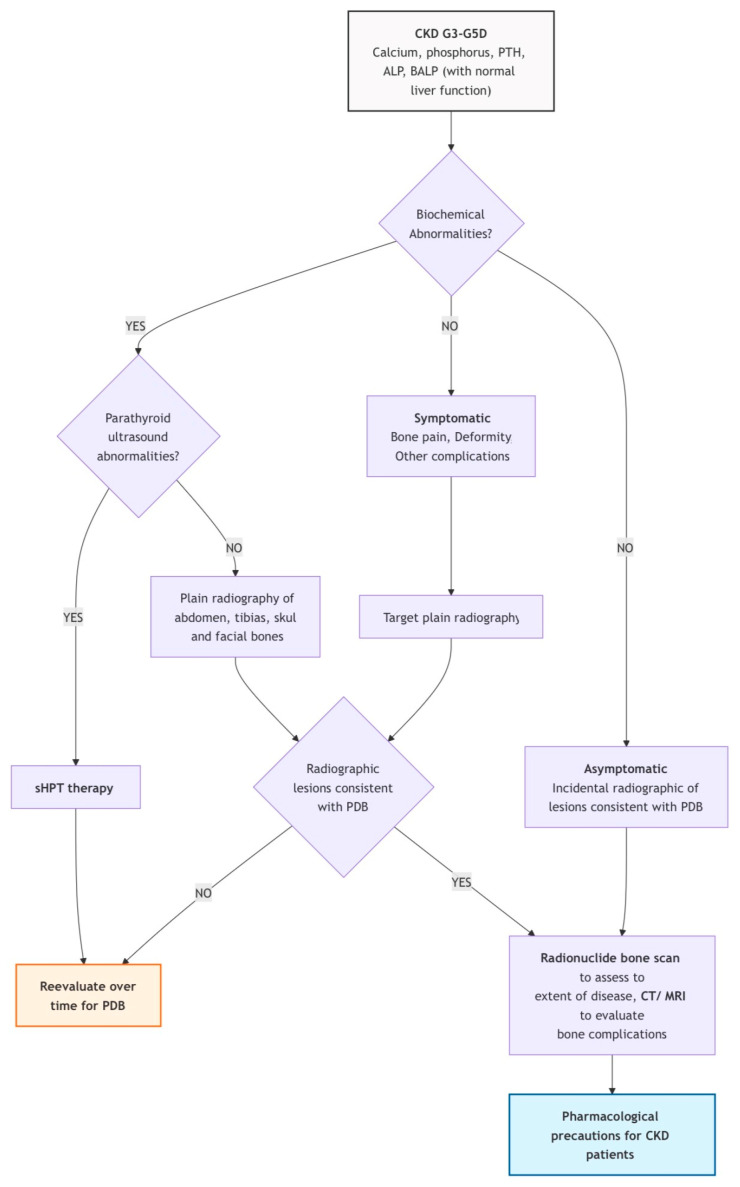
Diagnostic pathway for PDB in CKD patients. Legend: white rectangle = start of diagnostic process, diamonds = step requiring a decision, purple rectangles = phases of the diagnostic process, yellow and blue rectangles = end of the diagnostic process.

The following summary in Figure 3 is a hypothetical approach to identifying PDB-CKD by applying guideline suggestions valid for the general population to CKD patients. For patients with confirmed CKD-MBD, focusing diagnostic efforts on those with bone pain, high PTH, high ALP, and elevated blood calcium may be practical. This approach could reduce the need for extensive imaging studies. In fact, radiological methods are not mentioned among the useful diagnostic strategies for CKD-MBD [78]. However, asymptomatic cases or those with normal PTH and ALP levels may remain undetected. BALP determination could assist in identifying subgroups for further testing, though asymptomatic PDB in CKD patients without CKD-MBD signs may still go unrecognized. PDB can present at CKD diagnosis or emerge later. Thus, clinicians must consider both diseases concurrently throughout CKD management. The radiological alterations of PDB, being an expression of the various phases of bone remodeling, are only significant when detected in combination. Therefore, they can be identified in sufficiently extensive lesions, and the experience of radiologists is also important for a prompt diagnosis (as reported in one case) [65]. PDB assessment in CKD patients with valvular and vascular calcifications and kidney stones may be useful in addition to post-parathyroidectomy testing if PTH levels remain elevated. Furthermore, in the evaluation of patients with suspected or known PDB it is suggested to always evaluate the eGFR EPI formula in addition to the simple creatinine dosage to also highlight the early or moderate stages of CKD and to adopt the appropriate pharmacological precautions.

### Research Questions and Considerations in Relation to CKD-MBD and PDB

1. What influence might CKD-MBD have on PDB outcomes?
2. What influence could sHPT in CKD and nephrological therapy used for its control have on PDB outcomes?
3. Could even advanced uremic conditions alone impact PDB?
4. Does the coexistence of the two diseases worsen morbidity and mortality compared to the presence of only one disease?

## 13. PDB and Kidney Transplantation

The interaction between PDB and kidney transplantation is an underexplored area. The KDIGO guidelines for transplant candidates do not list PDB among the absolute or relative contraindications, and evaluations for kidney transplant suitability remain at the discretion of individual transplant teams [85]. A search on PubMed and Google Scholar revealed only one case report of a monostotic PDB patient deemed unsuitable as a living kidney donor. This patient was excluded from the transplant program due to concerns about potential kidney damage from painkillers and bisphosphonates, which may be required to manage PDB in the future (one case) [86]. This decision aligns with the precautionary principle, prioritizing donor and recipient safety during selection [87]. Donor selection criteria encompass not only autoimmune and chronic diseases but also other chronic conditions of uncertain origin [88]. The above criteria may make PDB a potential concern due to its unknown etiology and lack of curative treatment. Should this principle also be applied in the evaluation of transplant candidates with PDB? Most transplanted kidneys gradually lose function due to rejection, drug toxicity, renal artery stenosis, or ureteral obstruction [89]. The high frequency of NL found in PDB patients and related complications could also further reduce renal survival. Additionally, the association of PDB with hypertension and vascular and valvular calcifications could increase the already high cardiovascular risk of transplant recipients [90]. In fact, cardiovascular complications are the main cause of death in kidney transplant recipients [90]. In the absence of explicit guidance, the arguments presented above regarding the suitability of kidney transplant donors and recipients are based primarily on precautionary principles and isolated cases.

### Research Questions and Considerations in Relation to the PDB and Kidney Transplantation

1. How many patients eligible for kidney transplant have PDB?
2. How many kidney transplant patients have PDB?
3. If kidney transplant patients with PDB could be identified, would it be possible to evaluate the impact of CKD-MBD on PDB outcomes?
4. Could pre- and post-kidney transplant conditions be used to assess the impact of CKD on PDB outcomes? Could each patient’s post-transplant condition serve as a control group compared to the pre-transplant condition?
5. Do kidney transplant patients with PDB have worse morbidity and mortality outcomes than control groups?

## 14. Limitations

Questions, reflections, and hypotheses have emerged from the examination of this topic and could help in the management of patients suffering from PDB-CKD. Unfortunately, the available data do not allow for definitive conclusions to be drawn to support clinical practice. The most important information could be provided by longitudinal/cross-sectional and multicenter studies.

Currently, the few clinical cases summarized in Table 2 demonstrate the existence of patients with PDB-CKD, although they refer to distant settings and cover a 40-year period. The study by M. Kessler demonstrates that PDB-CKD cases can be identified through research in dialysis settings [67].

A 2024 Italian position paper on PDB provides no new developments compared to the guidelines mentioned in this text [91]. The 2024 KDIGO guidelines for CKD-MBD also refer to what was expressed in the previous 2017 paper [92].

Perhaps a different approach to the disease, based on the comparison with CKD and the lack of data on the subject, could provide useful input for exploring new avenues of research.

## 15. Conclusions and Future Directions

PDB is not a rare disorder; in the EU, a disease is classified as rare if its prevalence does not exceed 0.05% of the population [93]. Even the coexistence of PDB and CKD is not a rare phenomenon, given that the estimated prevalence in Italy is at least 0.063%. Despite this, information on the topic is rare. Because effective management of PDB depends on early diagnosis and appropriate treatment planning, active pursuit of a PDB diagnosis by both primary care physicians and nephrologists is encouraged. Furthermore, identifying PDB-CKD patients could allow for a sufficient number of patients to be reached for prospective studies. The chronic kidney disease (CKD) setting may be particularly well suited to exploring PDB because of the shared characteristics of the two conditions, including the reliance on common biochemical markers (e.g., PTH and ALP) for diagnosis and monitoring. Specific attention should be paid to therapeutic choices for patients with reduced glomerular filtration rates (GFRs) to avoid treatment complications. Further, screening for PDB in renal transplant donor and recipient evaluations could contribute to identifying additional cases, as could routine follow-up in transplant patients. Given the similarities of the two populations, sharing data on the association between PDB and CKD could advance our understanding of PDB etiopathogenesis and inform best practices in management. We do not know whether CKD can influence the progression of PDB, and if so, how. In this regard, follow-up of patients with PDB and CKD who have undergone kidney transplantation could provide interesting data on the pre- and post-transplant periods. Above all, and more immediately, attention to the coexistence of the two diseases could improve patient management with personalized choices based on their renal function.

## Figures and Tables

**Table 1:** Estimation of the prevalence of the coexistence of the two diseases with known prevalence under the assumption of independence.

P(A ∩ B) = P(A) P(B) Taken from https://stat.accmed.org A and B Independent Variables
P(A) = P(PDB) P(B) = P(CKD)
P(PDB) = 1% PDB prevalence in Italy
P(CKD) = 6.3% CKD prevalence in Italy
P(A ∩ B) = prevalence of coexistence of PDB and CKD in Italy
P(PDB ∩ CKD) = P(0.1) P(0.63) = 0.063%
Prevalence of coexistence of PDB and CKD in Italy = 0.063%

**Table 2:** Cases of PDB and CKD described in the literature. Taken from [53], updated and completed by L. Traversari.

Year	Author	No. of Cases	Gender/ Age	CKD/HD/ PD	M (Monostotic)/P (Polyostotic) Form	sHPT/Other Bone Diseases	Treatment of sHPT/ Parathyroidectomy	Symptomatic/ Asymptomatic	Treatment of PDB
1985	J.D. Ringe [54]	1	f/48	HD		sHTP+ aluminum- induced osteomalacia	parathyroidectomy	bone pain	
1998	R. Lorho [55]	1	m/83	HD	M/pelvis	sHTP	alfacalcidol and parathyroidectomy	undocumented	undocumented
2008	J. Estremadi [56]	1	f/77	HD	M/skull	no	no	bone pain	alendronate
2009	L. Wu [57]	1	f/77	PD	M/lumbar spine	undocumented	undocumented	radicular syndrome	calcitonin
2010	G. Cianciolo [58]	1	f/69	HD	M/skull	sHPT	sevelamer, cinacalcet, paricalcitol	undocumented	clodronate
2012	E. De Sousa-Amorim [53]	1	m/72	PD	P/right iliac bone, cervical spine, left ulna, external malleolus of left ankle	sHPT	cinacalcet, calcifediol	asymptomatic	no
2016	R. P. Kenneth [59]	1	m/68	HD (PBD was diagnosed 14–15 years before the start of dialysis)	P/pelvis, thoracic spine, right humerus, skull	sHPT	paricalcitol, calcium acetate, Lanthanum carbonate, ergocalciferol/calcitriol	undocumented	no
2018	N. Kuthiah [60]	1	f/63	CKD G4	P/left iliac and pubic bone, left tibia	undocumented	undocumented	symptomatic	denosumab
2019	PK. Chan [61]	1	m/80	CKD G5	M/pelvic bone	sHPT	undocumented	symptomatic	denosumab
2020	V.A. Panuccio [62]	1	m/60	CKD (PD was started 3 years later)	P/long bones, skull	sHPT	calcium-based phosphate binder, active Vit. D	bone pain	clodronate, calcitonin
2022	G. Elbuken [63]	1	m/47	CKD	P	undocumented	undocumented	undocumented	denosumab
2025	J. Hand [64]	1	m	HD	M	undocumented	undocumented	bone pain	pamidronate
2025	L. Traversari [65]	1	m/75	CKD G4-G5	P/skull, pelvis, long bones, sternum, clavicles, scapulae, rib arches, left humeral head	sHPT not present on arrival in the ward, occurred during follow-up	paricalcitol cinacalcet	bone pain	bisphosphonates, calcitonin, denosumab, calcitonin + vitamin D
2025	L. Traversari [65]	1	m/53	HD	p/left iliac bone, let femoral head, vertebrae	sHPT	calcium, active Vit. D, sevelamer	asymptomatic	no
2025	L. Traversari [65]	1	m/79	HD	P/right, iliac bone, right femoral head, chest	sHPT	active Vit. D, paricalcitol, sevelamer	asymptomatic	no
2026	D.G. Yavuz [66]	1	m/61	PD (started 4 years before)	m/right femur	sHPT	cinacalcet	asymptomatic	no bisphosphonate

Abbreviations: PD, peritoneal dialysis; HD, hemodialysis, CKD, chronic kidney disease; sHPT, secondary hyperparathyroidism; PDB, Paget’s disease of bone.

**Table 3:** Pharmacological precautions for CKD patients.

Drug	GFR	Recommendation
IV bisphosphonates	60 > GFR > 35 mL/min/1.73m^2^	space out the doses
IV or oral bisphosphonates	GFR < 35–30 mL/min/1.73 m^2^	generally contraindicated
NSAIDs	GFR < 60 mL/min/1.73 m^2^	advised against
NSAIDs + diuretics or RASIs	GFR < 60 mL/min/1.73 m^2^	advised against
Calcium and vitamin D supplements	GFR < 60 mL/min/1.73 m^2^	maintain normocalcemia

**Table 4:** Classification of renal osteodystrophy [83].

Types of Renal Osteodystrophy	High/Low Bone Turnover	Histological Picture
Osteitis fibrosa	High	Osteoclasts and osteoblast: increased number and activity Osteoid deposition: increased con woven pattern (most common) Peritrabecular fibrosis: variable amounts
Mixed uremic osteodystrophy	Hight and low	Osteitis fibrosa areas near osteomalacia areas
Osteomalacia	Low	Mineralization: low Osteoid deposition: increased and overlapping bone trabecula
Adynamic bone disease	Low	Mineralization: normal Osteoclasts and osteoblasts: reduced number and activity Osteoid deposition: reduced

**Table 5:** Indications currently available for the diagnosis of metabolic bone diseases [35,78,81].

Bone Disease	Biochemical Markers	X-Rays	DEXA (Dual Energy X-Ray Absorptiometry)	Biopsy
PDB	●	●		
CKD-MBD	●		* only in selected cases	* only in selected cases
Osteoporosis	▲		●	

● Diagnostic tests recommended by guidelines for individual conditions. ▲ To rule out secondary forms and evaluate bone metabolism. * In patients at high risk of fractures or a history of fragility fractures, if the outcome will impact treatment decisions.

## Data Availability

No new data were created or analyzed in this study.
